# Incorporation of Ions into Nanostructured Anodic Oxides—Mechanism and Functionalities

**DOI:** 10.3390/molecules26216378

**Published:** 2021-10-22

**Authors:** Anna M. Brudzisz, Damian Giziński, Wojciech J. Stępniowski

**Affiliations:** Institute of Materials Science and Engineering, Faculty of Advanced Technology and Chemistry, Military University of Technology, 2 Kaliskiego Str, 00908 Warsaw, Poland; damian.gizinski@wat.edu.pl

**Keywords:** anion incorporation, anodic oxides, nanopores, nanotubes, anodic aluminum oxide, anodic titanium oxide, photoluminescence, etching, catalysis

## Abstract

Anodic oxidation of metals leads to the formation of ordered nanoporous or nanotubular oxide layers that contribute to numerous existing and emerging applications. However, there are still numerous fundamental aspects of anodizing that have to be well understood and require deeper understanding. Anodization of metals is accompanied by the inevitable phenomenon of anion incorporation, which is discussed in detail in this review. Additionally, the influence of anion incorporation into anodic alumina and its impact on various properties is elaborated. The literature reports on the impact of the incorporated electrolyte anions on photoluminescence, galvanoluminescence and refractive index of anodic alumina are analyzed. Additionally, the influence of the type and amount of the incorporated anions on the chemical properties of anodic alumina, based on the literature data, was also shown to be important. The role of fluoride anions in d-electronic metal anodizing is shown to be important in the formation of nanostructured morphology. Additionally, the impact of incorporated anionic species, such as ruthenites, and their influence on anodic oxides formation, such as titania, reveals how the phenomenon of anion incorporation can be beneficial.

## 1. Introduction

**Figure 1:**
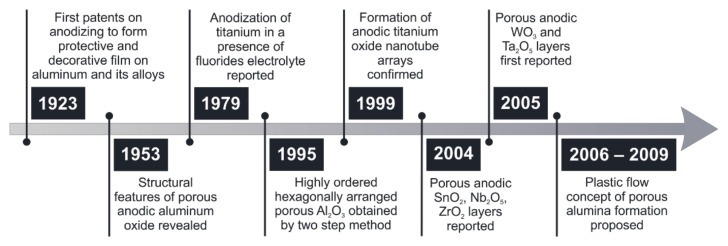
Timeline of the most relevant events in the history of anodizing of metals.

Anodization is a commonly used name for electrochemical oxidation of metals [1,2,3], their alloys [4,5] and semiconductors [6,7,8] under either galvanostatic or potentiostatic conditions in a two-electrode setup since the oxidized material plays a role of an anode. Originally, the major application of anodic oxidation was corrosion protection of lightweight aluminum alloys [9,10]. It was found that the electrochemically grown, uniform, compact, and insulating oxide layer formed on the alloy’s surface improved the adhesion of primer and paint, corrosion performance, surface hardness, and provided a high quality aesthetic of the treated surface (see Figure 1).

A fundamental change in the application of anodizing originated from the groundbreaking work of Masuda and Fukuda published in 1995 [11]. They reported for the first time a two-step anodization of aluminum, which leads to the formation of hexagonally arranged, honeycomb-like, and highly ordered anodic aluminum oxide (AAO). Anodizing pioneers revealed that the morphology of the prepared material (e.g., pore diameter and interpore distance) can be finely tuned by adjusting the operating conditions, including type, concentration and temperature of the electrolyte, as well as the applied anodizing voltage or current density [12,13]. Those findings prompted subsequent studies on the influence of other anodizing parameters on the AAO morphology [14,15,16] and allowed the development of various tools for pore arrangement quantification [17,18].

**Figure 2:**
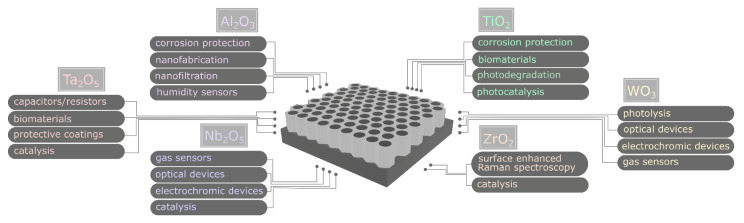
Most significant applications of selected nanostructured metallic oxides prepared by anodization.

An impressive amount of research on anodic alumina provides progress in nanofabrication [19] of materials with emerging applications—like biomimetic materials [20], CO_2_ conversion [21], energy storage [22], or superconductive materials [23]—and stimulates research on the anodization of other metals. The most significant applications of nanostructured oxides formed by the anodization of popular metals are presented in Figure 2.

For example, copper anodizing contributes to such important applications as CO_2_ electrochemical reduction [24], methanol fuel cells [25], photocatalytic water splitting [26] and microplastic decomposition [27]. Anodic titania is used in such important aspects as photocatalytic water splitting [28], hazardous compounds neutralization (e.g., Bisphenol A and Rhodamine B [29], or chromates [30]) and microplastic decomposition [31]. Moreover, anodic titania is also gaining the attention of researchers as a drug-releasing platform [32] and sensor [33]. Nanostructured anodic zirconia is also utilized as a photocatalyst [34] or as a substrate for Surface Enhanced Raman Spectroscopy (SERS) [35]. Additionally, other metals, like Hf [36], Mo [37], Nb [38], Sn [39], Ta [40], W [41,42], Zn [43,44], gain much attention as starting materials for anodizing.

Despite the countless amount of publications on applications of the nanostructured anodic metallic oxides, there are still certain fundamental aspects of anodizing that require deepened exploration. For example, there are still various, often contrary views on the mechanism of the anodic alumina growth [45]. Further development of more reliable tools is necessary for studying the initiation and evolution of the pores’ arrangement throughout the anodization [46]. Researchers are still exploring novel anodization regimes and new electrolytes [47,48] or combining various electrolytes to improve the quality of the formed oxides [49]. Challenges associated with the anodization of items with sharp edges made of aluminum and its alloys [50] as well as scaling-up of the process are still being studied [51].

Although numerous experimental and theoretical reports have been published on the topic of anionic species incorporation into the metallic oxides formed by anodization (mainly Al and Ti), this issue is typically only mentioned in review articles. The incorporation of electrolyte anions into the nanostructured anodic oxides is of particular interest, as it can influence multiple properties of the formed material, and provide interesting applications. Therefore, in this short literature review, the most relevant and interesting findings on anion incorporation during anodization of selected metals are showcased and discussed in detail. The most important question we want to answer in this review is which properties, and to what extent, can be modified by the electrochemical doping of anodic oxides with anionic electrolyte species, and hopefully inspire further progress in this field.

## 2. Incorporation of Electrolyte Anions into Anodic Aluminum Oxides

Anodization of aluminium, leading to the formation of anodic oxide (Al_2_O_3_), is the most widely studied [52]. Two types of anodic aluminum oxide can be formed, depending on the nature of the used anodizing electrolyte. A non-porous oxide is formed in neutral electrolytes [53]. Conversely, the porous anodic aluminum oxide is formed in acidic electrolytes, such as selenic, sulfuric, oxalic, phosphoric, chromic, malonic, tartaric, citric, malic acid, etc., in which the formed anodic oxide is slightly soluble [54].

The incorporation of the electrolyte-derived anions is considered a general phenomenon occurring throughout the formation of both the non-porous [55,56,57] as well as the porous anodic alumina [58,59,60]. A significant amount of work on the topic of incorporation of electrolyte ions into non-porous anodic oxide has been reported in the last decades of the 20th century [61,62]. Wood et al. [61] proposed a model of anion incorporation in non-porous aluminum oxide. The electrolyte anions from the double-layer adsorb at the aluminum oxide surface. During alumina film growth, the adsorbed anions are incorporated into the Al_2_O_3_. The electrolyte species can be immobile, migrate inward or migrate outward throughout the alumina under the electric field. The concentration of the incorporated species in the non-porous alumina film depends upon: (*i*) the type and concentration of the electrolyte; (*ii*) the direction of migration of the electrolyte species in the alumina; and (*iii*) the faradaic efficiency of the alumina growth. 

In the last few decades significant technological and scientific interest has been focused on the porous-type AAO due to its practical applications, e.g., for fabrication of membranes [63] and nanostructured materials [64]. For this reason, in this work we will focus only on more recent works about the incorporation of electrolyte species in porous anodic aluminum oxide and their influence on the properties of AAO, e.g., chemical properties (i.e., oxide solubility) [65,66], phase transition during calcination [67], capacitance [68], refractive index [69], and photoluminescence [70,71].

**Figure 3:**
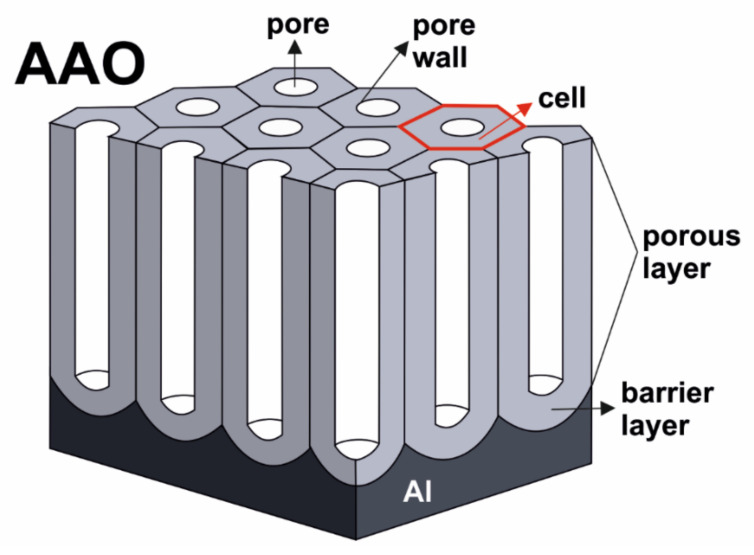
Schematic morphology of porous anodic aluminum oxide (AAO) on Al foil. Reproduced with permission from Ref. [64]. Copyright 2020 Elsevier Ltd.

From the morphological point of view, the porous anodic aluminum oxide is composed of two layers: the barrier-type layer (BL) and the porous-type layer. Figure 3 depicts an idealized structure of porous AAO. A large number of parallel, cylindrical pores—aligned perpendicularly to the aluminum substrate—forms a self-ordered hexagonal structure resembling a honeycomb. The bottom of each pore is closed by a hemispherical barrier layer of Al_2_O_3_. The aluminum oxide surrounding each pore (i.e., pore walls) constitutes a hexagonal cell. The parameters of the AAO, such as thickness, pore diameter, interpore distance, BL thickness can be easily controlled by adjusting the anodizing conditions [1,14]. It is important to point out that the oxide cells are self-organized in a highly ordered, hexagonally arranged structure only when proper anodizing conditions (i.e., anodizing regimes) are used. Finally, the surface of aluminum is textured with an ordered array of concaves formed during anodization, corresponding to the morphology of the barrier layer. Interestingly, those concaves can be used to govern the pore arrangement during the second anodization and improve the ordering of formed AAO morphology [11]. 

### 2.1. Mechanism of Anions Incorporation: Duplex and Triplex Structure

**Figure 4:**
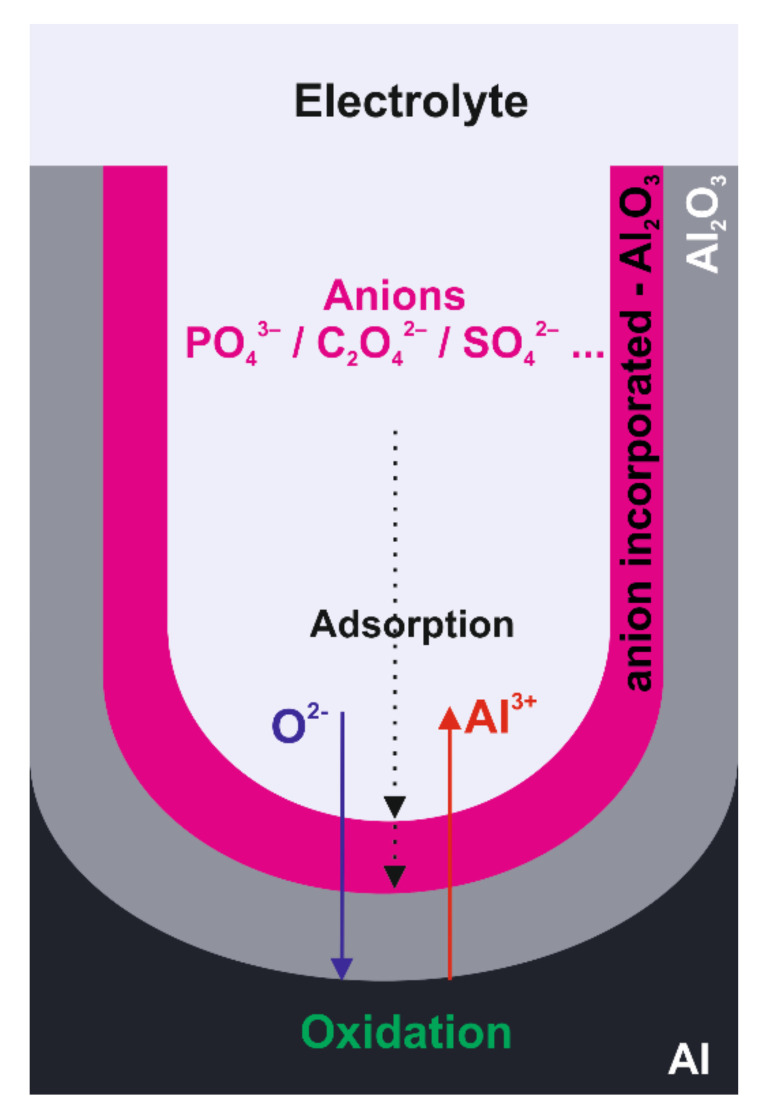
Schematic representation of the mechanism of AAO formation and anion incorporation.

The mechanism of anion incorporation in the porous anodic alumina was proposed as follows (see Figure 4.) [53,72]. 

During the anodization of aluminum substrate, part of the Al^3+^ anions are formed at the metal/oxide interface (Equation (1)):

Al → Al^3+^ + 3e^−^ (1)

Subsequently, those anions drift through the oxide layer due to the electric field and are ejected into the solution at the oxide/electrolyte interface. At the same time, oxygen-containing ions (like O_2_^−^ or OH^−^) migrate from the electrolyte bulk through the oxide layer to the metal/oxide interface, resulting in the oxidation of aluminum. The electrolyte anions (i.e., conjugated base anions), formed due to the dissociation of the applied acidic electrolyte in water solution, can be adsorbed at the pore bottom/electrolyte interface, and substitute OH^−^ or O^2−^ in the oxide. All anions are pulled towards the positively charged electrode by the electric field [55,56,73]. However, due to larger size and lower mobility as compared to OH^−^ or O^2−^, their migration velocity is much lower. As a result, electrolyte anions concentration will decrease from the sidewall outer to inner layers. 

**Figure 5:**
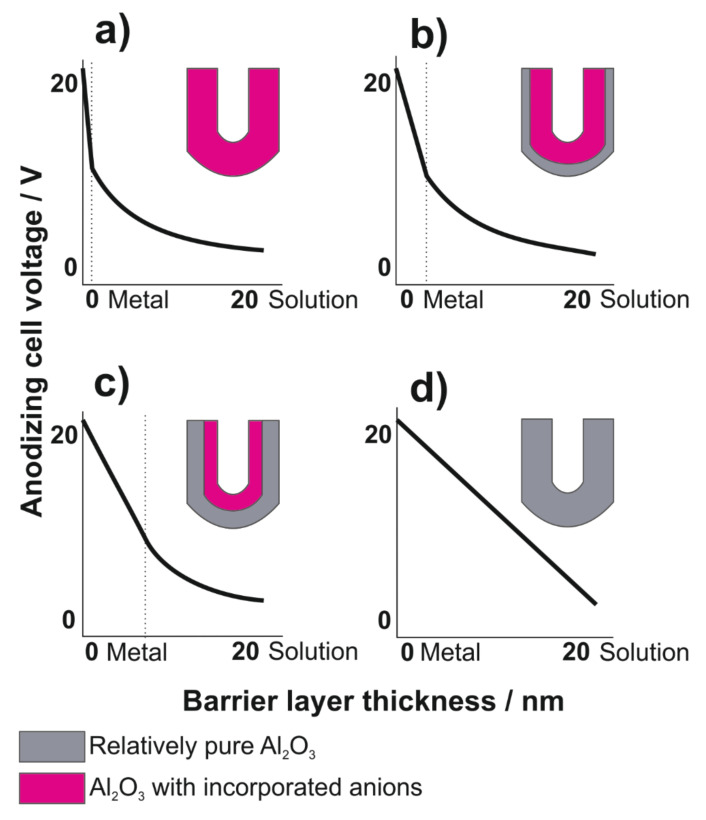
Distribution of the potential drop and electric field, E (slope of the voltage−distance plot), across barrier layers of porous AAOs formed in (**a**) sulfuric, (**b**) oxalic, (**c**) phosphoric, and (**d**) chromic acid. Reproduced with permission from Ref. [1]. Copyright 2014 American Chemical Society.

Indeed, the experimental work revealed that the anion incorporation of the most commonly used anodizing electrolytes, i.e., oxalic, sulfuric and phosphoric acid (Figure 5a–c), occurs via inward migration under an electric field during the anodization of aluminum [60,74,75,76]. Moreover, no uniform distribution of incorporated ions across the oxide layer was observed. Thompson et al. in a series of papers [53,60,61,77] proposed and discussed a model of the duplex structure of the cell walls. Two different regions—the inner layer containing relatively pure alumina and the outer layer with incorporated electrolyte anions—were distinguished. It was also revealed by Thompson and Wood [78] the thickness of the relatively pure layer depends on the type of electrolyte used during anodization. 

Thompson and Wood [78] related the steady-state anodizing growth of porous AAO films formed in the most common anodizing acids to the distribution of the acid anions within the barrier layer and the true field strengths across the relatively pure alumina regions. The electric field applied to the aluminum oxide during its growth is inhomogeneous: higher in the inner layer, and lower in the outer layer, i.e., the incorporated anions reach layer. In other words, they correlated the thickness of the anion-free layer with the potential drop across the barrier layer during anodization. For a given voltage, the larger the thickness of the anion-contaminated outer layer, the higher the electric field strength at the inner layer and the larger the oxide growth rate [61]. The potential drop (ΔU) is greater and linear across the relatively pure alumina region and smaller across the outer acid anion-contaminated region, where the potential decreases progressively towards the aluminum oxide/electrolyte interface (Figure 5). Choi et al. [79] reported that the duplex layer exists not only in the pore walls but also in the barrier layer. The thickness of the outer oxide layer in the barrier is exactly the same as that in the wall. However, the inner oxide layer in the center of the hemisphere of the BL is twice as thick as that in the wall, while the inner oxide at the edge of the hemisphere is the same as that in the wall. 

Fukuda and Fukushima [80] proved that the distribution of the SO_4_^2−^ ions in the pore walls depends on the electric field. The duplex structure was confirmed for oxalic acid, where the existence of the C_2_O_4_^2−^ anion impurities was proved [74,81]. Ono et al. [82] studied the structure of pore cell walls formed during the anodization of aluminum in 0.4 M phosphoric acid. The duplex structure of the pore walls was confirmed for samples formed at anodizing potential higher than 10 V. 

The nature of anions incorporated during the anodization of species has been also studied. Yamamoto and Baba [83] utilized electron spin resonance (ESR) and infrared (IR) spectroscopy combined with a chemical sectioning technique to study the nature of oxalate species incorporated into porous anodic alumina films. IR and X-ray photoelectron spectroscopy (XPS) measurements have shown that electrolyte species incorporated into the growing oxide are mostly anions formed by the hydrolysis of acids or salts added to the electrolyte [61]. It was postulated that the species incorporated during anodization are coming from the electrolyte, i.e., PO_4_^2−^, C_2_O_4_^2−^ and SO_4_^2−^, while the elemental distribution of P, C and S follow a bell-like (parabola-like) distribution along the barrier layer, across the pore wall, and along the pore wall [84,85]. Their concentration at the oxide surface is significant and increases with oxide thickness. Its value becomes a maximum inside the oxide at the distance increasing with oxide thickness and this value becomes highest at an intermediate thickness, at least for the carbon and sulfur. Then, their concentration decreases and becomes almost zero at the aluminum oxide/metal interface as well as at cell-wall boundaries. The distribution of incorporated anions in pore walls and the barrier layer is in agreement with theoretical models calculated by Mirzoev et al. [86,87]. A special case of anodizing in chromic acid is characterized by the absence of incorporated anions (Figure 5d). It has been shown that chromate anions are accumulated at the outer oxide surface and do not penetrate into the oxide body [88].

It is generally acknowledged that the number of incorporated acid anions and their distribution (i.e., depth) in the walls of anodic alumina depend strongly on the conditions of anodization, i.e., type and concentration of the used electrolyte, applied cell voltage (U) or current density (j), and temperature (T) [89,90]. Additionally, the content and depth of anionic impurities incorporation decrease as a function of the anodization duration, due to the progressive reduction of the electrolyte concentration [65]. Typical concentrations of species incorporated into the porous anodic alumina are 12–14 wt.% for sulfate, 6–8 wt.% for phosphate, and 2.4 wt.% for oxalate [53]; however, even higher concentrations of SO_4_^2−^ have been reported [66].

Each type of layer in the duplex structure of the AAO has a different dielectric constant. When compared to the dielectric constant of the pure alumina, the anion-incorporated alumina has a lower dielectric constant. In other words the more anions are incorporated in the alumina, the lower the dielectric constant. Moreover, the outer oxide layer has a nonhomogeneous effective dielectric constant depending on the concentration of impurities [79]. As the anion contamination decreases towards the inner oxide layer and the outermost oxide layer, the effective dielectric constant for both regions increases, which is in agreement with the discussed duplex structure of AAO.

The duplex structure has been reported for other electrolytes, e.g., malonic acid [91,92], sodium hydrogen sulfate solution at various concentrations [93], phosphonic acid (H_3_PO_3_) at 150 V [94] as well as in 0.3 M arsenic acid (H_3_AsO_4_) at 320 V [95]. Takenaga et al. [96] studied anion incorporation in AAO formed during anodizing in 1.0 M etidronic acid at 215 V and 25 °C for 1 or 2 h. This molecule has two phosphorous atoms and two carbon atoms in the molecular structure; however, the distribution of P and C atoms in AAO originating from the anions was clearly different. The duplex structure was observed for phosphorus; however, the carbon was distributed uniformly in the oxide. The authors suggested that the chemical bonds of incorporated anions with a large molecular structure are cleaved in the alumina during anodizing. Further research is therefore necessary for better understanding the incorporation of larger anions, as the resulting properties are of particular interest. For example, AAO formed in etidronic acid is more resistant to etching in 2.5 M NaOH, and pore sealing in boiling water is slower as compared with AAO prepared in the same conditions in sulfuric acid [97].

**Figure 6:**
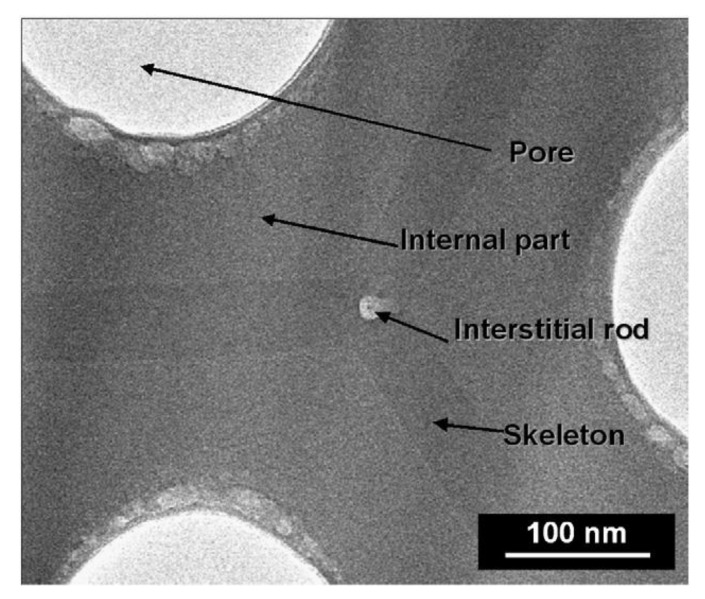
TEM plane view of AAO-PA, prepared in 8 wt.% phosphoric acid, at constant applied voltage of 185 V for 4 h and −1.5 °C, showing the different parts of the pore wall (i.e., the outer pore wall, cell-boundary band, and interstitial rod). Reprinted with permission from Ref. [98]. Copyright 2009 Elsevier Inc.

A triplex structure of the cell walls (Figure 6) was reported for AAO formed by anodization conducted in a 8 wt.% phosphoric acid solution (AAO-PA) at 185 V for 4 h at −1.5 °C [98]. Additionally, in the typical outer and intermediate layers contaminated by electrolyte species, pure alumina “interstitial rods” with a diameter of about 14 nm at the intersection of the tree hexagonal cells were observed. Since the size of the phosphate anion complex is larger than the size of the other anions, the adsorbed PO_4_^3−^ migrates more slowly as compared with other anions [55]. The phosphate anions are delayed in the intermediate part since the attracting force in the intermediate part is weaker than that in the electrolyte/oxide interface. As a result, PO_4_^3−^ is concentrated in the intermediate part of the outer wall. 

As an interesting example, anodization in pyropohospohoric acid should be mentioned, which leads to the formation of honeycomb oxide with nanofibers [99,100]. The electron energy loss spectroscopy (EELS) revealed that phosphorus was incorporated into the barrier layer; however, the nanofibers were composed of relatively pure alumina, equivalent to the previously reported “interstitial rods”. The formation of the above-mentioned morphology was a result of the higher solubility of anion-contaminated AAO in the anodizing electrolyte, as compared to the typically used anodizing electrolytes. From the applicative point of view, the resulting material exhibited superhydrophilic behavior. 

A method for purposefully implanting anion species in pore walls was provided by Patermarakis et al. [85]. The authors discussed the processes controlling anion incorporation at the barrier layer/double layer interface. The presence of cations with similar mobility to Al^3+^ in the anodizing electrolyte favors a high concentration of aluminum cations and anions in the double layer. This causes a higher rate of anion incorporation, and in turn, leads to a lower dissolution rate of pore walls in the electrolyte during anodizing. It results in lower pore diameter and, consequently, formation of AAO with lower porosity. 

In summary, electrolyte anion incorporation is an inherent and intrinsic aspect of porous anodic alumina formation. It influences not only the morphology of the obtained material, but also the composition. Of more importance are its physical and chemical properties, which are discussed below. 

### 2.2. Properties of the AAO Related to Incorporated Anions

#### 2.2.1. Refractive Index

The optical properties of AAO are closely related to its composition, and therefore the anion’s incorporation. For example, the control of the refractive index of nanoporous anodic alumina layers is an important step towards the development of devices in optical and chemical sensors as well as in biosensing. Minguez-Bacho et al. [69] studied the variation of the refractive index of nanoporous AAO films as a function of the concentration of incorporated sulfate anions. The performed calculations were based on an iterative method combining Snell’s law and constructive interference conditions for thin films. The variation of the refractive index of 0.08 with the sulfur incorporation was constant within the 400–1200 nm range of the reflectance spectra. The values are higher for the lower amounts of sulfate anions embedded into the nanoporous AAO films, which corresponds to values closer to the refractive index of pure alumina. Fan et al. [101] pointed out that increasing annealing temperature during the heat treatment of AAO would influence its refractive index by decreasing the concentration of oxalic impurities due to their thermal decomposition.

#### 2.2.2. Photoluminescence and Galvanoluminescence

Another property of AAO oxide attributed to incorporated anions is an intense and broad photoluminescence band in the blue region when excited under ultraviolet light (UV). Despite a large number of publications on this topic, the exact origin and mechanism of AAO photoluminescence are still debatable [102]. The PL can be modulated by the pore diameter, the variation of excitation wavelength and the change of the annealing temperature [103]. In the literature, there are two competing theories regarding the origin of photoluminescence in anodic aluminum oxide. The first one considers that the defect centers related oxygen vacancies (F^+^) induce the PL of AAOs, and the PL intensity is influenced by the F^+^ center’s concentration. The second one points out that oxyacid radical intermingled in AAOs during electrochemical anodizing and, consequently, gives rise to the PL properties.

In 1981, Yamamoto et al. [104] proposed that the oxalate anions incorporated in AAO can be transformed into luminescent centers, showing a blue photoluminescence band around 470 nm. As a dihydric acid, oxalate presents delocalized π bonds which would be responsible for excitation in the ultraviolet region. According to Vrublevsky et al. [105], the anion incorporation during the anodization is a result of two simultaneous reactions: water electrolysis and dissociation of a hydrogenoxalate anion (HC_2_O_4_^−^) that results in the incorporation of oxygen and oxalate (C_2_O_4_^2−^) anions inside the oxide structure [16,25]. Vrublevsky et al. [105] also suggested that when Al is anodized in an oxalic acid solution, the formation of a bidentate coordination compound based on carboxylate ion and Al^3+^ ions occurs. The emission is related to structural defects in AAO coexisting in both, oxide and barrier layers. In these alumina films, there are two emission centers: F center and F^+^ centers, which were characterized as color emission centers. F centers correspond to an oxygen vacancy, and the charge in F^+^ represents ionized vacancies. These can be considered as charged point defects that might form energy levels inside the optical band gap of the oxide, acting as active recombination centers when the material is excited by light in the UV region. Under a steady electric field during anodization, OH^−^ will gradually migrate to the oxide/metal interface, transform into O^2−^ and form new alumina, and the anions in electrolyte solution will drift to the anode [106]. The anions will either trap one or two electrons and form F+ or F centers, respectively. Anions that trap one electron are always dominant—hence the PL band intensity of F+ centers is always greater than F centers. This particular explanation was utilized by multiple authors studying the photoluminescence of AAO prepared in oxalic acid [107] or sulfuric acid [108] or their mixtures [109]. In the latter publication, the authors pointed out that a Xe lamp, commonly used as an excitation source, is not proper to study the emission of samples prepared in sulfuric acid.

The Rutherford Backscattering Spectrometry (RBS) analysis performed by Vrublevsky et al. [105] did not reveal a presence of incorporated carbon in AAO formed by galvanostatic anodization. It was assumed that contrary to potentiostatic anodization, under the galvanostatic regime, oxalate anion incorporation is not favorable. Conversely, the incorporation of impurities could occur from water electrolysis. Multiple authors agreed that high-intensity emission bands could be related to the presence of a high concentration of oxygen vacancies [70,101,102]. 

Canelli et al. [102] studied the photoluminescence of nanoporous anodic alumina prepared by galvanostatic anodization by applying 5 mA cm^–2^ for 90 min at 20 °C in three different electrolytes: oxalic acid, phosphoric acid, and an equimolar mixture of oxalic and phosphoric acids. After anodization, the Al substrate was removed from the AAO porous layer in a solution of CuCl_2_ and HCl. Depending on the used electrolyte, the incorporation of impurities was different due to the imposed constant current density regime, leading to different concentrations of oxygen vacancies inside the AAO. The authors did not observe the incorporation of oxalate ions, which is typically attributed to being the origin of PL emission. However, the PL spectrum of the AAO membranes prepared in oxalic acid displayed the most intense emission band in the blue region when excited with UV energy as compared to other impurities [102].

On the other hand, Fan et al. [101] studied the incorporation of oxalates, i.e., C_2_O_4_^2−^ in porous aluminum oxide formed by anodization in 0.3 M oxalic acid, under a constant voltage of 40 V, at 10 °C for 5 min. After removal of the Al substrate and annealing in vacuum for 3 h at 400–900 °C infrared vibrational measurements revealed the existence of C_2_O_4_^2−^ impurities in the AAO. The COO^−^ absorption band intensity decreased with increased annealing temperature, due to decomposition of the impurities related to oxalate anions [101]. Xu et al. [70] showed that during annealing of AAO prepared by anodization in oxalic acid, the incorporated anions undergo decomposition into either CO_2_ or carboxylic groups. At temperatures above 800 °C stable carboxylic impurities are formed, e.g., aluminum-carboxylate complex [104]. 

Vrublevsky et al. [110] studied the photoluminescence of anodic alumina membranes prepared by anodization in 0.4 M tartaric acid at constant current density of 60 A m^–2^ and temperature of ca 18 °C. The partially oxidized hydrogenated amorphous carbon (α-C:H) clusters were formed during anodization in tartaric acid, as confirmed by Fourier-transform infrared spectroscopy (FTIR). They are linked to the oxide network through the carboxylate ions and coordinative polyhedra of Al^3+^ ions. The optical gap measured for annealed AAO was 3.25–3.5 eV, which is much lower than for pure alumina (6.5 eV).

**Figure 7:**
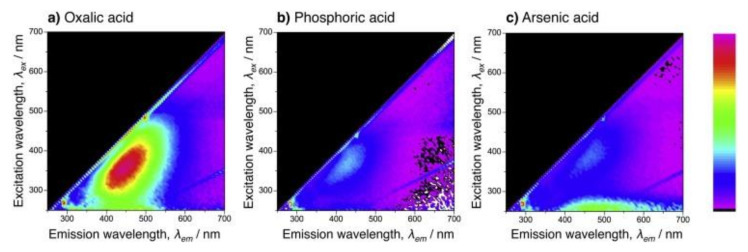
Excitation-emission-intensity maps of the porous alumina formed by anodizing in (**a**) oxalic acid, (**b**) phosphoric acid, and (**c**) arsenic acid solutions. The scale to the right indicates the relative intensity of the photoluminescence emission. For the oxalic and phosphoric acid anodizing processes, the electropolished specimens were anodized in 0.3 M electrolyte solutions (293 K) at a constant current density of 20 A m^−2^ for 2 h. Reproduced with permission from Ref. [95]. Copyright 2017 Elsevier B.V.

The AAO prepared by anodization in 0.3 M arsenic acid (at 0 °C, 320 V for 72 h) exhibited no photoluminescence emission under UV irradiation at 365 nm and a white hue under UV irradiation at 254 nm [95]. Under the latter irradiation, an intense photoluminescence emission band with a center wavelength of approximately 515 nm was measured on the porous alumina. Figure 7 exhibits excitation-emission-intensity maps of the porous alumina formed by anodizing in (a) oxalic acid, (b) phosphoric acid, and (c) arsenic acid solutions [95]. In the map for porous alumina formed by oxalic acid anodizing (Fig. 2.4a), a strong photoluminescence emission was identified in the 300–450 nm range in excitation and the 400–500 nm range in emission. Porous alumina films formed by organic carboxylic electrolytes such as oxalic and malonic acid exhibit a blue photoluminescence emission.

In the case of phosphoric acid anodizing (Figure 7b), an extremely weak peak was measured at approximately 370 nm in excitation and 430 nm in emission, and no visible photoluminescence emission was observed. In contrast, a characteristic broad peak (250–280 nm range in excitation and 400–700 nm range in emission) was measured from the porous alumina formed by arsenic acid anodizing (Figure 7c). The white hue from the porous alumina corresponds to this broad photoluminescence emission with visible regions of 400–700 nm. The white photoluminescence emission may be due to the arsenate anion vacancies incorporated from the electrolyte solution during anodizing. However, the difference between the effect of phosphate (PO_4_^3−^) and arsenate anions (AsO_4_^3−^) on the photoluminescence behavior is still not clear; therefore, further investigation is required. The use of arsenic acid may be limited to particular applications such as a closed system due to their toxicity. 

Interestingly, other anionic species incorporated in the AAO during anodization can influence the photoluminescence. Stępniowski et al. [111,112,113] studied photoluminescence of anodic alumina with an incorporated copper/ethylenediaminetetraacetic acid complex ([CuEDTA]^2−^), indigo carmine or vanadyl citrate chelate anions, respectively. The AAO with incorporated [CuEDTA]^2−^ exhibited PL emission bands at 280 and 320 nm (excitation 250 and 275nm). The indigo carmine shifted the PL emission bands to 650 and 661 nm (excitation at 600 nm). The AAO doped with vanadyl citrate chelate anions exhibited photoluminescence bands at 270 and 455 nm when a Xe lamp was used as the source. The conducted research not only confirmed that foreign anions (i.e., not serving a role of electrolyte) from the electrolyte can be incorporated into anodic alumina, but can be used to influence the measured photoluminescence. 

Stojadinovic et al. [114] studied galvanoluminescence (GL) of AAO formed by galvanostatic (in the range from 5 mA cm^–2^ to 10 mA cm^–2^) and potentiostatic (in a range from 80 V to 120 V) anodization in 0.25 M malonic acid aqueous solution. The authors observed wide GL (measured at 450 nm) bands in the visible region of the spectrum and two dominant spectral peaks. The first one was almost independent of the anodizing voltage and situated at about 455 nm. The second spectral peak shifted from about 530 nm (for applied anodizing voltage of 80 V) to about 580 nm (up to an applied voltage of 120 V). The relative ratio of the second and first spectral peaks increased with the applied cell voltage. Moreover, the position of luminescence intensity peaks and their relative ratio depended on the temperature of the electrolyte. According to the authors, the same shape of measured spectra and correlation with anodizing voltage for both electrolytes indicated that the same types of GL centers are responsible for galvanoluminecence in organic electrolytes. The GL spectra obtained for AAO prepared inorganic electrolytes (phosphoric and chromic acid) were different, suggesting different GL mechanisms.

#### 2.2.3. Chemical Properties and Application as Humidity Sensors

The AAO membranes prepared by etching of the residual Al and subsequent barrier layer removal are widely used for nanofabrication [115]. The controlled removal of the barrier layer is particularly important. Although several methods have been developed, wet-chemical etching is widely used for this purpose. Wet-chemical etching is a gradual dissolution of anodic alumina in 5 wt.% phosphoric acid solution. Although this technique is very prominent in the literature, the number of publications on the influence of the chemical composition (i.e., composition and depth of incorporated impurities) on the rate of Al_2_O_3_ etching is limited [65,116]. As it was mentioned before, the chemical composition of AAO is influenced by anodization conditions, e.g., type of electrolyte, its concentration, applied voltage, current density, etc. To produce AAO membranes in a more reliable and reproducible manner, the barrier oxide layer etching process should be more carefully controlled and re-optimized if anodizing conditions are changed. The ability to precisely control the diameter of the pores is a particularly attractive feature of AAO as a template for nanofabrication. It provides a tool to systematically investigate the size dependence of chemical or physical properties of ordered arrays of nanodots, nanowires, or nanotube materials prepared using porous AAO templates.

Han et al. [65] studied the effect of oxalic acid concentration during anodization on the barrier oxide etching behavior. They observed that the anions incorporated in the AAO strongly influence the rate of Al_2_O_3_ dissolution during wet-chemical etching, both in the barrier layer as well as in the pore walls. The authors showed that pore wall oxide is etched at a higher rate (1.04 nm min^−1^) in the early stage than in the later stage (0.36 nm min^−1^). The slowed rate of wet-chemical etching in the later stage can be attributed to the relatively pure nature of the inner pore wall oxide, as compared to the less dense outer pore wall oxide due to the incorporation of anionic species. The barrier oxide removal time was found to be longer for thicker AAO layers (i.e., formed by longer anodization). According to secondary ion mass spectrometry (SIMS) analysis, a lower level of anion impurity content was incorporated into the barrier oxide layer of AAO formed by long-term anodization, as compared to those formed by short-term anodization. Moreover, the etching rate of the outer pore wall at the top part of porous AAO was found to be higher as compared to the bottom part of AAO. Those observations indicated the formation of a gradient of impurity concentration along the pore axis. Han et al. [65] attributed this effect to both (i) continuous decrease of electrolyte concentration and (ii) disordering of pores occurring due to the decreased current density during long-term anodization. 

He et al. [117] studied the AAO prepared by anodization in 0.3 M oxalic acid as humidity sensors. The anions incorporated into the AAO influence the electronic and ionic surface conductivities of the AAO film, as well as capacitance. Therefore, exposing more anions to the surface by pore widening using wet-chemical etching in a phosphoric acid solution enhanced the sensitivity of the sensor at low humidity. Conversely, at high humidity, the permittivity constant increased due to the high density of surface ions.

#### 2.2.4. Phase Transitions during Annealing

In order to improve the mechanical strength, flexibility, and resistance to chemical attack, heat treatment of the AAO membranes is used [118]. On the one hand, the most thermodynamically stable α-alumina gained scientific attention for the production of light-emitting diodes or sapphire glass. On the other hand, γ-alumina was used as a catalyst for hydrogen production. The incorporated anions affect the phase transition of AAO during heat treatment, as shown by Cho et al. [119] on the basis of ^27^Al magic-angle-spinning nuclear magnetic resonance (MAS NMR) and FTIR. An X-ray diffraction (XRD) analysis revealed that the structure of AAO fabricated in oxalic acid (AAO-OA) was amorphous at or below 800 °C, and it changed to γ-alumina at 850 °C. At higher temperatures, as the heat treatment temperature was increased, a coexistence of γ- and δ-alumina phases was observed in the 900–1000 °C range; however, at 1050 °C the existence of only δ-alumina was detected. Interestingly, in the range of 900–1000 °C, a characteristic decrease of mass was observed, attributed to the decomposition of oxalate species [118]. Finally, at 1100 °C, a coexistence of δ- and α-alumina phases was observed. For AAO fabricated in phosphoric acid (AAO-PA), an amorphous structure appeared at or below 800 °C, as was the case with AAO-OA. However, only δ-alumina existed in the 850–1100 °C range. It was concluded that the incorporated impurities underwent different thermochemical reactions during annealing. In the case of AAO-PA, an AlPO_4_ structure with a chain shape of “Al-O-P-O-Al” was formed and was not removed during a high-temperature heat-treatment process.

## 3. Ion Incorporation during Anodization of Other Metals

Ion incorporation during anodization of other metals, such as tantalum, niobium, titanium and iron, has also been investigated throughout recent decades. A controllable transferring of ions from the electrolyte to the oxide layer grown during anodization of the above-mentioned metals can serve as a facile method for tuning their photo-electronic properties and/or steering the morphology of the obtained materials. 

### 3.1. Titanium Anodization in Fluorides Containing Electrolytes

Particularly, anodization of one of these metals has been drawing considerable interest over recent decades and it has been intensively studied since the 1980s. This metal is titanium, which, when subjected to anodization, can form self-organized oxide tube or pore arrays. Such a uniform morphology that can be designed by applying proper conditions, together with the excellent photocatalytic features of grown TiO_2_, makes Ti anodization a very important field of electrochemical surface modification. In 1979, Kelly et al. [120] published their work, in which the influence of fluorides’ presence in the electrolyte on titanium passivity was studied. Even though the high-resolution electron microscopy investigation of the formed oxide layer was lacking, the author concluded that TiO_2_ nanoporous morphology was obtained. Two decades later, in 1999, Zwilling et al. [121,122] showed, for the first time, a self-organized anodic nanotube layer grown during Ti anodization in chromic acid electrolyte with the addition of hydrofluoric acid. It was found that the applied anodization conditions led to the formation of a 500 nm thick oxide layer moderately organized in a nanotube array. The key finding was the recognition that F^−^ ions are crucial for obtaining this self-organized morphology.

#### 3.1.1. Field-Assisted Ejection Theory

**Figure 8:**
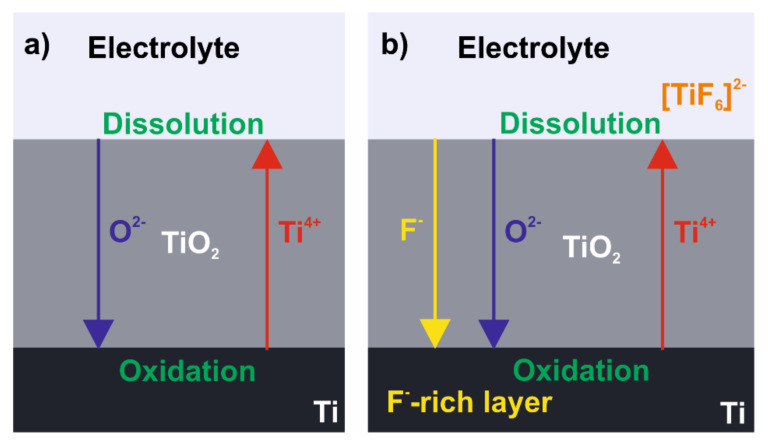
Schematic representation of oxide layer formation on titanium during anodization in (**a**) electrolyte without addition of fluoride ions and (**b**) fluoride ions containing electrolyte.

Currently, titanium anodization is usually conducted with electrolytes containing 0.1–1 wt.% fluoride ion concentrations in the potential step procedure at a constant voltage up to 30 and 150 V for aqueous and non-aqueous electrolytes, respectively. A highly ordered hexagonal array of nanotubes in the TiO_2_ passive layer was found to be successfully formed in organic electrolytes, such as ethylene glycol [123], ionic liquids [124], protic solvents [125] or by adapting a two-step anodization procedure that was originally reported for generating a porous anodic layer of alumina [126,127]. In all cases, however, the presence of fluoride ions is required for obtaining self-ordered nanopores or nanotubes morphology. When titanium is subjected to anodization in an electrolyte without fluoride ions, only a compact oxide layer is attained. Growth of the layer proceeds as Ti^4+^ species are formed and migrate from the metal surface towards the bulk of the electrolyte. Simultaneously, O^2−^ ions are generated in field-assisted deprotonation of H_2_O or OH^−^ and migrate towards the metal surface as illustrated in Figure 8a. The mobility of ionic species through the growing oxide layer undergoes field-aided transport, and the rate at which both Ti^4+^ and O^2−^ migrate determines where the oxide is formed. Under most experimental conditions, the O^2-^ migration rate is significantly higher than for Ti^4+^, and therefore oxide is grown at the metal–oxide layer rather than the oxide–electrolyte interface.

To affect the constant formation of the compact oxide layer during Ti anodization, fluoride ions need to be introduced in a sufficient concentration. On the one hand, when fluoride ions stand for less than 0.05 wt.% of the electrolyte, the oxide layer grows as in the case of fluoride’s absence in the system, i.e., compact. However, above this value, fluorides start to interact with Ti species in a twofold manner: (i) fluorides react with Ti^4+^ at the oxide–electrolyte interface leading to the formation of water-soluble [TiF_6_]^2^^−^ as represented by Equation (2); (ii) fluorides chemically attack grown TiO_2_ (see Equation (3)).

Ti^4+^ + 6F^−^ → [TiF_6_]^2−^ (2)

TiO_2_ + 6F^−^ + 4H^+^ → [TiF6]^2−^ + 2H_2_O (3)

On the other hand, when fluoride concentration exceeds ca. 1 wt.%, all of the released Ti^4+^ are consumed and intensive complexation prevents growth of the oxide. Thus, a suitable concentration of fluorides in electrolytes for nanostructured titania coating is estimated to be in a range of 0.1–1 wt.%. In this range growth, the oxide competes with Ti^4+^ ejection at the oxide–electrolyte layer and oxide erosion by F^−^ attack. As a consequence, a porous oxide layer is formed (Figure 8b). In a general mechanism of titania layer growth with an intermediate concentration of fluorides, the first stage of the process is as in a fluoride-free medium, hence, a compact oxide layer is developed. This can be observed as a current drop in the *I–t* curve registered during anodization. In a consecutive stage, as oxidation continues, highly irregular nanopores appear due to F^−^ attack. As a consequence, current increases since the surface of the reactive area develops. Another current drop occurs as nanopores start to organize, assembling in a regular pattern. Eventually, longer anodization leads to the steady growth of tubes and current density stabilizes at a constant value [128,129,130].

In the field-assisted ejection theory for Ti anodization, the presence of fluorides inhibits the formation of a compact titania layer by chemical etching of the oxide and solvation of Ti^4+^ migrating towards the electrolyte. These phenomena maintain a relatively thin layer of oxide that subsequently can be arranged into a nanoporous pattern. Another important outcome that needs to be taken into consideration when discussing the titanium anodization mechanism in the fluoride-containing electrolyte is the formation of a fluoride-rich layer near the metal–oxide interface. Since the F^−^ migration rate through the oxide layer is significantly higher than for O^2−^, fluorides can easily penetrate the growing oxide and accumulate underneath it [131]. The presence of this fluoride-rich layer formed by F^−^ incorporation is the basis for another concept that explains the mechanism for TiO_2_ nanotube arrays’ formation during anodization: plastic flow theory.

#### 3.1.2. Plastic Flow Concept

**Figure 9:**
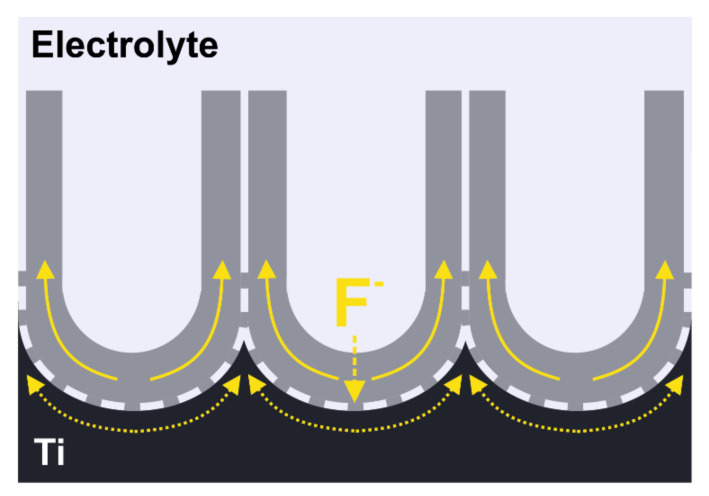
Conceptual representation of plastic flow of viscous oxide that leads to formation of nanotubular patterns during Ti anodization with fluorides.

In 2006, Thompson et al. [132,133], and a few years later Hebert et al. [134,135], proposed and modeled the flow concept for the formation of porous alumina. As it was proposed, volume expansion and electrostrictive forces occurring during oxide growth induce compressive stresses. Accordingly, in the high electric field, the oxide barrier layer is pressed against the metal surface causing ionic movement near the metal–oxide interface as the film gains plasticity. As a result, a viscous oxide is compressed and flows through the tube walls towards the oxide–electolyte interface leading to tube elongation (see Figure 9) [136].

The ratio of the molar volume of the grown oxide to the molar volume of the consumed metal during electrooxidation can be represented by the Pilling–Bedworth ratio (PBR) [137]. This factor defines volume expansion in the process and its value implies valid conclusions regarding the growth mechanism studied in anodization. Generally, PBR can be correlated to the current efficiency of the process, and its value changes as oxide formation proceeds [1]. It is expected since any morphological transformations, such as pore formation, are observed as changes in current curve evolution during anodization.

For compact barrier-type TiO_2_ layer formation (no fluoride in the system), PBR was found to be 2.43 [138]. Berger et al. [139] investigated how PBR differs for three consecutive stages of Ti anodization in a fluoride-containing electrolyte. In the initial phase (0–70 s), when the compact layer is formed irrespectively of fluoride presence, PBR was estimated to be 2.4, and the value confirms the previously reported data. Successively, when stage II is initiated and current density increases as pores start to form in oxide film (70–200 s), PBR decreased to 1.7~1.9, suggesting that a significant part of the metal was released to the electrolyte as a water-soluble [TiF_6_]^2−^ species. However, in the third stage (200–3600 s), when self-organization of the tube array occurs, PBR was found to be 2.7~3.1. This value was higher than PBR for compact layer formation, which implied an additional mass transport phenomenon that reorganizes oxide distribution in the film without increased metal consumption. It was noted that in the case of PBR ≥ 2.4 for Ti anodization, TiO_2_ nanotube formation follows the plastic flow mechanism. 

Recently, similar conclusions were reported by Zhu et al. [140,141]. The authors conducted a series of experiments for Ti anodization in a fluoride-containing medium with a particular focus on the volume expansion ratio and its normalization by current efficiency. For compact barrier-type titania layer formation with a PBR of ca. 2.4, current efficiency would amount to 100%. If the volume expansion ratio exceeded this value (such as PBR = 2.7~3.1 for stage III of nanotubes formation), it would mean that current efficiency should be higher than 100%. Nevertheless, it was noted that real current efficiency is reduced to below 100% due to the presence of a fluoride-rich layer on the metal–oxide interface created by mobile F^−^ ions incorporation [142]. The authors revealed in their study that the volume expansion ratio, normalized by current efficiency, is also higher than the value expected by field-assisted ejection theory. Therefore, different nanotubes formation mechanisms should be considered.

**Figure 10:**
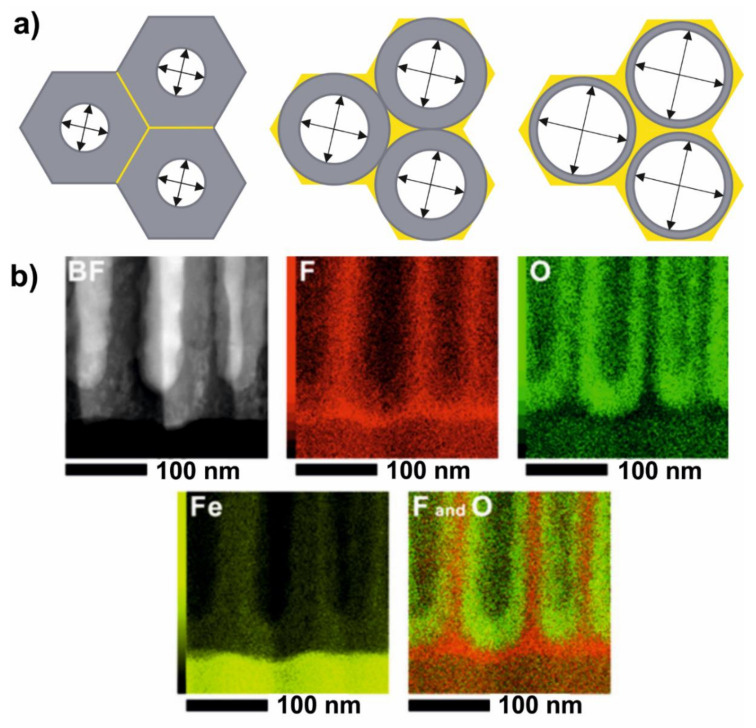
Nanotubes array formation during anodization in fluoride containing electrolyte: (**a**) top view of fluorides distribution in tubes array; (**b**) atomic resolution microscopic analysis of fluoride-enriched layer of anodized iron. Reproduced with permission from Ref. [143]. Copyright 2018 John Wiley and Sons.

Undoubtedly, fluorides incorporation into the growing oxide during titanium anodization plays a crucial role in the formation of self-organized arrays. In the plastic flow concept, the fluoride-enriched interlayer, similarly to the oxide film, gains plasticity and both phases are flown outwards in the same configuration through the walls of the pores. Accumulated fluoride ions are transported in an ionic movement towards the oxide–electolyte interface and their release output to the electrolyte is located at triple points of hexagonally arranged pores (Figure 10a). Taking into account the fact that fluorides create water-soluble species with titanium, it can be expected that in a water-containing medium, hollow spaces separating individual tubes are formed. Therefore, the presence of fluorides and water in the electrolyte is a key factor during Ti anodization for obtaining the nanotube’s morphology rather than the nanopore’s.

The presence and the role of the fluoride-enriched layer in nanotube pattern formation in the anodization process was shown by Habazaki et al. [143]. Researchers studied the oxide growth mechanism during iron anodization in a NH_4_F-containing electrolyte. A thorough investigation of grown nanotubular and nanoporous oxide films with atomic resolution analytical electron microscopy allowed detecting and mapping fluoride ions in obtained coatings. As shown in Figure 10b, the distribution of species involved in the process clearly indicates an accumulation of fluorides in a layer underneath, forming tubes. Furthermore, as plastic flow reorganizes oxide morphology, fluorides are transported through cell boundaries outwards to the electrolyte. It was confirmed that water concentration in the ethylene glycol electrolyte impacts the morphology of the oxide, as FeF_x_ species are released out of a wall’s boundaries from the solid film. The nanotube’s morphology was more distinguished as water concentration increased; therefore, the transition from a pore to a tube structure can be steered by tuning the water content in the electrolyte.

### 3.2. Incorporation of Transition Metals Species

Nanostructured TiO_2_ prepared by facile and scalable anodization method finds a wide range of applications in numerous technological fields. It offers a highly organized morphology that can be easily tailored by adjusting anodization conditions and electrolyte composition. One exceptional feature which makes TiO_2_ one of the most studied compounds in material science is its electronic structure. Titania in crystal form is a semiconductor with a broad bandgap (E_g_ < 3 eV) and, therefore, can be applied in solar cells and photocatalytic reactions. 

The anodization method also enables the introduction of another modification to grown titania films by supportive ionic species incorporation. Electrochemical doping of TiO_2_ with transition metals (incorporated as oxyanions) or nonmetallic anions can be used for tuning its photoelectronic properties. Kernazhitsky et al. [144] performed a comparative investigation to study the influence of transition metals doping on photocatalytic properties of anatase and rutile forms of nanocrystalline TiO_2_. It was found that those two titania forms are affected by various metals cations impurities in different manners and to different extents. Dopants such as cationic species of Fe and Cr introduced significant changes to the electronic properties of anatase by narrowing its bandgap by ca. 0.1 eV and consequently increasing its potential photocatalytic activity. The same modification in the rutile structure had an almost negligible impact on its bandgap since it was broadened by only 0.01 eV. 

In another study, Choi et al. [145] demonstrated how Ru can be incorporated into the TiO_2_ oxide layer during anodization for modifying its activity in O_2_ and Cl_2_ evolution reactions. For that, ruthenium-containing salt (KRuO_4_) was added to the electrolyte in small amounts with concentrations ranging from 0.002 to 0.0002 M. Oxyanions of transition metals can be incorporated into the growing oxide layer as field-assisted migration of negatively charged species. It was reported that the applied one-step anodization procedure allows Ru incorporation into nanostructured titania and the amount of Ru in oxide strongly depends on the applied potential. As investigated with XPS depth profiling, the greater the applied potential, the higher the amount of incorporated Ru in anodized samples. This trend was valid only in the case of potential lower than 60 V. Therefore, the greatest amount of Ru was incorporated into the titania film at 60 V and at higher potentials, a high density of the oxide-forming layer inhibited the diffusion of RuO_4_^−^ ions. The presence of Ru in TiO_2_-based materials was found beneficial for its catalytic performance in O_2_ and Cl_2_ evolution from 1 M NaOH and 1 M HCl solutions, respectively. Electrodes prepared at an applied cell voltage of 60 V with the highest content of ruthenium indicated significantly lower overpotentials in both reactions when compared to bare TiO_2_. In consecutive studies, Choi et al. [146] enhanced the efficiency of Ru incorporation to nanostructured titania by a two-step anodization procedure. They reported a method for short shock treatment at a high applied potential (up to 200 V) in KRuO_4_ containing electrolytes of pre-anodized titanium. Subjecting pre-prepared TiO_2_ electrodes to such harsh conditions for a few seconds resulted in a significant increase of Ru incorporation (ca. 5 at.%). As previously reported, similar conclusions were made in this case. There was an optimal potential that balances layer density and amount of incorporated Ru species. Samples prepared at 140 V shock treatment indicated the best catalytic performance by lowering the onset potential and increasing current density in the oxygen evolution reaction to the highest extent in the conducted study. 

Using a similar approach, Rohani et al. [147] performed multi-incorporation of C, N and Ni into nanotubular titania via anodization in a K_2_[Ni(CN)_4_]-enriched electrolyte. Multiple characterization techniques allowed determining the presence of incorporated species that can act as photoactive sites. The optimized anodization procedure enabled the incorporation of N atoms to the TiO_2_ lattice as N-Ti-O or N-Ti-N and C atoms as carbonates—Ti-O-C. The presence of Ni in the dopant led to the substitution of Ti atoms in the oxide lattice and introduced oxidized Ni species to the system. Comparison between undoped TiO_2_ and modified material revealed significant improvement of the photoactive properties after modification. N, C and Ni incorporation led to narrowing the bandgap of TiO_2_ and an extended absorption spectrum in the visible light range, which consequently enhanced the photoactive efficiency of doped electrodes for applications such as water splitting. 

Incorporation of cationic dopants in a form of cyanides and oxyanions was widely employed in recent years to substitute Ti^4+^ ions in anodically grown TiO_2_ and consequently improve the photo-efficiency of the material. Examples of anodization procedures and applications of various doped TiO_2_ nanostructures are collated in Table 1.

### 3.3. Incorporation of Nitrogen to Other Metals 

Tuning TiO_2_ photoactivity in UV and visible regions by incorporation of non-metallic anionic species such as C, F, N, S, B or P was investigated and reported in recent years [155,156,157,158]. However, C and N incorporation attracted the most attention due to the considerable improvements of TiO_2_ photoelectronic features [159,160]. In the case of carbon incorporation, C atoms substitute O species in the TiO_2_ structure, which introduces new energy levels above the TiO_2_ valance band. Therefore, the bandgap of titania is narrowed down and a shift of adsorption spectrum is observed [161].

Substitutional N incorporation was calculated to be the most effective of the above-mentioned anionic dopants in bandgap narrowing from mixing N *2p* states with O *2p* states in titania [162,163]. Its presence was proven to have a great impact on visible light harvesting efficiency by shifting the absorption spectrum to lower energy regions [164], as well as leading to changes in electrical conductivity, reflective index or increased hardness [165,166]. Nitrogen is the most widely used dopant for improving TiO_2_ photoelectronic properties; however, its incorporation during the anodization process is not obvious. Nevertheless, it was reported that it is possible to incorporate N into oxide coating during anodization when nitrates [167] or ammonium ions [168] are present in the electrolyte. The introduction of N in the form of NH_4_^+^ ion carriers in an anodization process seems unintuitive and hardly possible, since, in the electric field, only anionic species can migrate inwards from the oxide layer that is formed on the anode. However, Ono et al. [169] observed that when the pH of the electrolyte during niobium anodization was raised to 10 by the addition of NH_4_OH, nitrogen was incorporated into the oxide film. In this work, the researchers were investigating the influence of the electrolyte’s pH on Nb anodization and found that nitrogen incorporation was highly dependent on this factor. XPS depth profiling revealed that changing the pH of the electrolyte from acidic to basic (pH = 10) allowed nitrogen to penetrate ca. 70% of the oxide layer. The presence of nitrogen in a Nb-based anode significantly enhanced its dielectric properties. Relative permittivity was doubled for the sample anodized in pH = 10 (i.e., 80.5) when compared to a sample obtained in an acidic medium (i.e., 43.7). The authors stated that there is a mechanism allowing nitrogen incorporation from ammonium ions, but it was not provided in the report.

A few years later, Habazaki et al. [170] reported similar observations for tantalum anodization. Nitrogen was found only in a sample that was subjected to electrochemical oxidation when the pH of the ammonium-containing electrolyte was 9, and nitrogen was located only in the outer layer of the oxide. Additionally, in this report, a mechanism for nitrogen incorporation was proposed to explain pH dependence on the film’s composition. It was stated that the electric field-assisted deprotonation process of OH^−^ on the oxide/electrolyte interface which yields O^2−^ is also responsible for the deprotonation of NH_4_^+^ to generate NH_3-x_^x−^ moieties. This form of ammonia ions can migrate into an oxide film towards the metal surface and react with Ta^5+^, which flows through the oxide film in the opposite direction. The pH of the electrolyte is a key factor for this mechanism to occur, because NH_4_^+^ ions must reach the oxide’s surface to undergo deprotonation. When the pH of the electrolyte is higher or lower than the isoelectric point (pH_pzc_), the oxide’s surface is expected to be negatively or positively charged, respectively, due to acid/base equilibria. Therefore, NH_4_^+^ deprotonation and, subsequently, NH_3-x_^x−^ incorporation is possible only when the pH of the electrolyte is higher than the pH_pzc_ of the formed oxide. The isoelectric point for Nb and Ta oxides equals 4.1 and 2.7 [171], respectively. Hence, it was possible to incorporate nitrogen into oxides’ structures in the above-mentioned cases. 

Furthermore, since nitrogen is highly electronegative, NH_3-x_^x−^ moieties can continue to lose protons as N-H bonds are weakened due to hydrogen bond formations in aqueous media [172]. The dissociation energy of the N–H bond in ammonia is lower than the dissociation energy of O–H in water (388 kJ mol^–1^ and 463 kJ mol^–1^, respectively); thus, field-assisted deprotonation may lead to the formation of both NH_2_^−^ and N^3−^ under anodization conditions. Considering that the radii of those N ionic species are comparable to O^2−^ and OH^−^ [173], it is possible to incorporate nitrogen from ammonia-containing electrolytes under suitable anodization conditions. 

## 4. Summary and Conclusions

Anion incorporation during anodizing of metals plays an important role in the formation of anodic oxides, influencing the resulting morphology. The models of anion incorporation in anodic alumina and titania were discussed in detail. Both the refractive index and the rate of chemical etching of contaminated Al_2_O_3_ depend on the concentration of anionic species in the oxide layer. Moreover, the incorporated anions lead to the emergence of unique features, such as photoluminescence and galvanoluminescence of anodic alumina. It is important to point out that not only electrolyte anions can be incorporated, as other species were successfully introduced into alumina during anodization, e.g., copper/ethylenediaminetetraacetic acid complexes, indigo carmine or vanadyl citrate chelate anions. It can be expected that the intentional incorporation of the designed species to influence selected properties of oxides could be a future field of research. Furthermore, the incorporation of other elements into anodic titania was found to be extremely beneficial in such applications as photocatalytic water splitting, as well as electrochemical O_2_ and Cl_2_ evolution. Therefore, anion incorporation was found to be one of the methods of the nanostructured anodic oxides electrochemical doping that can bring advances in emerging applications.

## Figures and Tables

**Table 1:** Recent developments in TiO_2_ doping with transition metals species in anodization of Ti.

Material Composition	Electrolyte Composition ^1^	Application	Reference
Fe(N, S)-TiO_2_	*DMSO*^2^, HF, *K_2_[Fe(CN)_6_]*	stainless steel corrosion protection	[148]
Fe-TiO_2_	EG ^3^, H_2_O, NH_4_F, *K_3_Fe(CN)_6_*	photodegradation of methylene blue	[149]
WO_2_-TiO_2_	DMSO, HF, *Na_2_WO_4_*	water splitting	[150]
W(S)-TiO_2_	EG, NH_4_F, H_2_O, *Na_2_WO_4_, K_2_S_2_O_7_*	water splitting	[151]
Cr-TiO_2_	EG, NH_4_F, H_2_O, *K_2_Cr(SO_4_)_2_*	stainless steel corrosion protection	[152]
Cr-TiO_2_	EG, NH_4_F, H_2_O, *K_2_CrO_4_*	water splitting, stainless steel corrosion protection	[153]
Mo(N)-TiO_2_	EG, NH_4_F, H_2_O, *K_2_MoO_4_*	photocatalysis	[154]

^1^ component written in *italics* stands for dopant source; ^2^ dimethyl sulfoxide; ^3^ ethylene glycol.
